# Shorter Infantile Amnesia in Females: Important Implications for the Next Generation †

**DOI:** 10.3390/cells14050354

**Published:** 2025-02-28

**Authors:** Yuheng Yang, Yuya Sakimoto, Makoto Goshima, Dai Mitsushima

**Affiliations:** 1 Department of Physiology, Yamaguchi University Graduate School of Medicine, Yamaguchi 755-8505, Japan; olliveryang@gmail.com (Y.Y.); ysaki@yamaguchi-u.ac.jp (Y.S.); b905eb@yamaguchi-u.ac.jp (M.G.); 2 The Research Institute for Time Studies, Yamaguchi University, Yamaguchi 753-8511, Japan

**Keywords:** hippocampal learning, sexual differentiation, infantile amnesia, testosterone, estrogen

## Abstract

The sex-specific development of hippocampal learning in juveniles remains unclear. Using an inhibitory avoidance task, we assessed contextual learning in both sexes of juvenile rats. While sex hormone levels and activating effects are low in juveniles, females showed superior performance to males, suggesting that females have a shorter period of infantile amnesia than males. It was already known that when infants are cared for by mothers with high parenting behavior, they are likely to become high parenting mothers themselves. In addition, neonatal testosterone is known to masculinize the brain, causing behavioral, neural, and hormonal sex differences. Here, we reviewed the purposeful significance of sex-specific development for learning, along with the interaction of developmental changes in the hormonal environment.

## 1. Introduction

Learning and memory are fundamental cognitive processes that are influenced by biological and environmental factors [1]. Several sex-regulated differences have been identified in the cognitive functions that acquire, store, and retrieve memory information. Previous studies have shown that genetic factors, hormonal milieu, and the development patterns of the brain are the cause of sex differences in cognitive abilities [2,3,4]. In fact, hippocampal acetylcholine (ACh) release, which is necessary for contextual learning and synaptic plasticity [5], is about 30% higher in males than in females, showing a sex difference in learning performance [6]. Contextual learning requires associative environmental stimuli such as a dark box and foot shock (aversive stimuli), and, by studying the brain in childhood, it is possible to elucidate the developmental process of sex-specific cognitive functions and neural mechanisms.

The inhibitory avoidance (IA) task is a widely used behavioral paradigm for assessing contextual memory. This task compares the latency of avoidance behavior before and after the experience of a foot shock. Previous studies have reported that adult male rodents outperform adult females in fear-conditioning tasks [6,7]. These findings suggest that sex differences in contextual learning may be under the influence of sex-specific neural circuitry or hormonal milieu.

The sex hormones in the brain, estrogen and testosterone, are known to influence hippocampal development and synaptic function. Estrogen has been shown to enhance synaptic plasticity, promote long-term potentiation (LTP), and support hippocampal-dependent learning and memory tasks [8,9,10]. On the other hand, testosterone and its metabolites also promote neuroplasticity but may have different effects depending on the developmental stage and brain region [11,12,13]. Since puberty (secondary sexual characteristics) is a period that produces dramatic differences in the hormonal milieu, and since we recently found several critical periods of hippocampal function in juvenile males [14], we focused here on sex differences in juveniles.

In brain development, neonatal exposure to testosterone is known to masculinize the brain. By administering testosterone to female newborns, they can acquire high male levels of hippocampal acetylcholine release and dramatic behavioral changes [15]. The effect of neonatal testosterone is known as an organizing effect, and the animals acquire male-specific properties in later stages of life [15]. In the hippocampus, a brain region important for learning and memory, there are differences in structure and function between males and females [16,17,18]. Testosterone in males or estrogens in females is known to maintain learning functions, dendritic spine density, synaptic connections, and changes in gene expression patterns.

Despite numerous studies, sex differences in contextual learning during the early postnatal period remain unclear. Previous studies have reported sex differences in contextual learning between postnatal day 24 (PN24) and PN29–36 [7,19], but evidence from earlier developmental periods is lacking. We have recently shown that the critical period for learning is PN16–17 [14], when the hormonal milieu of sex steroids is low and stable.

The present study aims to investigate sex differences in contextual learning during preadolescence using the IA task. Specifically, we focused on PN16 and PN17, the final phase of infantile amnesia, to investigate sex differences in learning function. By blocking the effect of neonatal testosterone, we can investigate the role of neonatal testosterone in contributing to sex differences in the critical period of contextual learning.

## 2. Materials and Methods

### 2.1. Animals

Pregnant Sprague-Dawley rats were obtained from Japan SLC, Inc (Hamamatsu, Japan). After delivery, both male and female pre-weaned rats were used in this study. The total number of studied animals and their body weights are summarized in Table 1. Rats were kept with their mother and littermates in opaque plastic cages (dimensions: 25 cm height, 25 cm length, 40 cm width) lined with wood chips. Housing conditions were maintained at a constant temperature of 24 ± 1 °C with a 12 h light/dark cycle (lights on from 8:00 a.m. to 8:00 p.m.). We counted the day of birth as postnatal day 0 (PN0), and litters were reduced to 10 pups per cage on day 2 (PN1) to ensure that each mother had sufficient resources for nursing. Additionally, each female was used for no more than three litters, with at least one month between litters to minimize potential litter effects. Water and food (MF, Oriental Yeast Co., Ltd., Tokyo, Japan) were provided ad libitum. All animal handling and experimental procedures adhered to the guidelines set by the Institutional Animal Care and Use Committee of Yamaguchi University. Ethical approval for this study was obtained from the Institutional Animal Care and Use Committee of Yamaguchi University Graduate School of Medicine (Approval No. 04-S02). These practices comply with the Guide for the Care and Use of Laboratory Animals published by the National Institutes of Health (NIH Publication No. 85-23, revised 1996).

### 2.2. Inhibitory Avoidance (IA) Task

**Figure 1:**
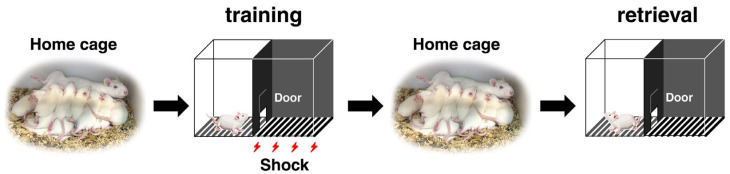
Schematic representation of the inhibitory avoidance (IA) task and experimental design. The illustration outlines the setup and procedure used for the IA task to evaluate learning and memory in juvenile male and female SD rats. Rats were housed with their mother and pups during the experiment. During the task, each rat was placed in the brightly lit compartment of a two-chamber apparatus. The apparatus consisted of a lit “safe” side and a dark “shock” side, separated by a door. Rats received a mild electrical shock upon entering the dark side, and 30 min later, the latency to enter the dark compartment was recorded as a measure of learning performance. Male and female rats were tested separately to assess sex differences.

The hippocampus-dependent IA task was conducted using a two-chamber apparatus designed to differentiate safe and shock areas. The box measured 33 cm in height, 33 cm in length, and 58 cm in width, and it was divided into a brightly lit “safe” compartment and a dark “shock” compartment by a trap door (Figure 1) [5].

During training, each male and female rat was gently placed in the illuminated side of the box, facing away from the trap door to standardize the starting position. Once the door was opened, the rats were allowed to freely explore and enter the dark compartment. The time it took for the rats to enter the dark area was recorded as the latency prior to IA learning. This latency served as a measure of exploratory behavior prior to the application of any aversive stimulus.

When a rat entered the dark chamber, the trap door was promptly closed to confine the animal, and an electrical foot shock (1.6 mA, 2 s) was delivered through metal rods embedded in the floor. The rats remained in the dark chamber for 10 s after the shock, ensuring consistent post-shock exposure across all subjects. Following this, the rats were carefully returned to their original home cage with their mother and pups.

Thirty minutes following the training procedure, each male and female rat was returned to the brightly lit compartment of the apparatus to evaluate memory retrieval. The time taken for the rat to re-enter the dark chamber was recorded as the post-training latency, serving as a measure of its contextual learning performance.

### 2.3. Statistical Analysis

Data and statistical analyses were performed using GraphPad Prism (version 10.2.0; GraphPad Software LLC, San Diego, CA, USA), Microsoft Excel (Microsoft Co., Redmond, WA, USA), and StatView software (version 5; SAS institute Inc., Cary, NC, USA). Statistical comparisons were made using two-way ANOVA, with sex as the between-group factor, and training as the within-group factor. Post hoc one-way ANOVAs were also performed for specific comparisons. *p* values < 0.05 were considered statistically significant.

## 3. Results

### Sex Differences in Contextual Learning Performance

**Figure 2:**
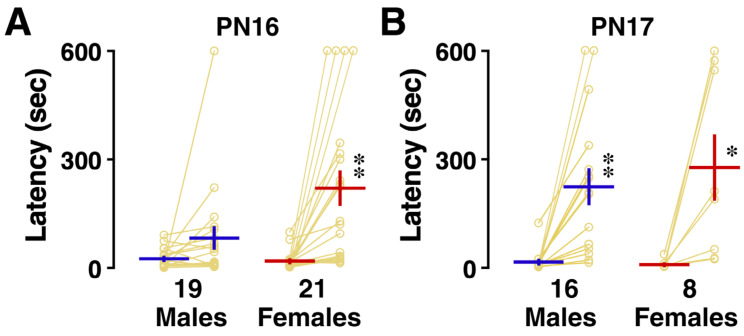
Sex differences in contextual learning performance. The left and right sides of the connected lines represent the latency before and after the foot shock. The latency to enter the dark compartment is presented as a measure of contextual learning performance. (**A**) At PN16, female rats showed significantly longer latencies compared to male rats, indicating superior learning ability (** *p* < 0.01). (**B**) At PN17, male rats showed a significant increase in latency, suggesting improved learning ability. In contrast, female rats consistently showed longer latency (* *p* < 0.05, ** *p* < 0.01). Statistical analysis was performed using two-way ANOVA repeated measures, followed by post hoc comparisons. Error bars represent the standard error of the mean (SEM), and individual data points (yellow circles) are connected to visualize within-subject changes. The number of animals is shown at the bottom of each graph.

The latency to enter the dark compartment during the IA task revealed significant sex differences and IA task effects (Figure 2). At PN16, a two-way ANOVA showed the main effect of sex (F_1,38_ = 4.842, *p* = 0.034) and training-induced change (F_1,38_ = 19.393, *p* < 0.0001). There was also a significant interaction (F_1,38_ = 6.038, *p* = 0.019). Post hoc one-way repeated measures ANOVA showed that male rats failed to learn the task (F_1,18_ = 3.104, *p* = 0.095) while female rats successfully learned the task (F_1,20_ = 17.994, *p* = 0.0004). Post hoc one-way factorial ANOVA showed that females had a longer latency than males after (F_1,38_ = 5.522, *p* = 0.024) but not before training (F_1,38_ = 0.711, *p* = 0.405), indicating superior contextual learning in females at this age.

At PN17, a two-way ANOVA showed a training-induced change (F_1,22_ = 26.467, *p* < 0.0001). However, neither the main effect of sex (F_1,22_ = 0.232, *p* = 0.635) nor the interaction was significant (F_1,22_ = 0.429, *p* = 0.519). Although the interaction was not significant, a post hoc one-way repeated measures ANOVA showed that both sexes of rats successfully learned the task (males: F_1,15_ = 18.239, *p* = 0.0007; females: F_1,7_ = 9.228, *p* = 0.019). Post hoc one-way factorial ANOVA showed no sex difference before (F_1,22_ = 0.416, *p* = 0.526) and after training (F_1,22_ = 0.325, *p* = 0.575).

## 4. Discussion

### 4.1. Developmental Changes in Learning and Memory

Although the learning function is immature in infants, the progressive increase in latency from juveniles to adults indicates a natural development of learning [14]. We then identified a critical day for learning at PN17 in males [14], but females successfully learned the task at PN16 (Figure 2). These results suggest an earlier functional maturation of hippocampal circuitry in females, and the critical day for learning in females is still unknown. Although cyclic changes in estrogen levels strengthen synaptic connections and establish the circuits that support hippocampal-dependent tasks in mature females, estrogen levels are low in juvenile females, so the contributing factors are unknown [20,21].

While hippocampal function develops steadily in females, the neonatal organizing effects of testosterone masculinizing the brain may delay maturation in males. Since testosterone levels are low, androgen receptor-mediated processes for both contextual learning and spatial navigation would not be available [22,23]. However, when males experienced the powerful activating effect of testosterone, adult males outperform adult females in hippocampus-dependent tasks [6].

### 4.2. Role of Steroid Hormones in Sex-Specific Learning

**Figure 3:**
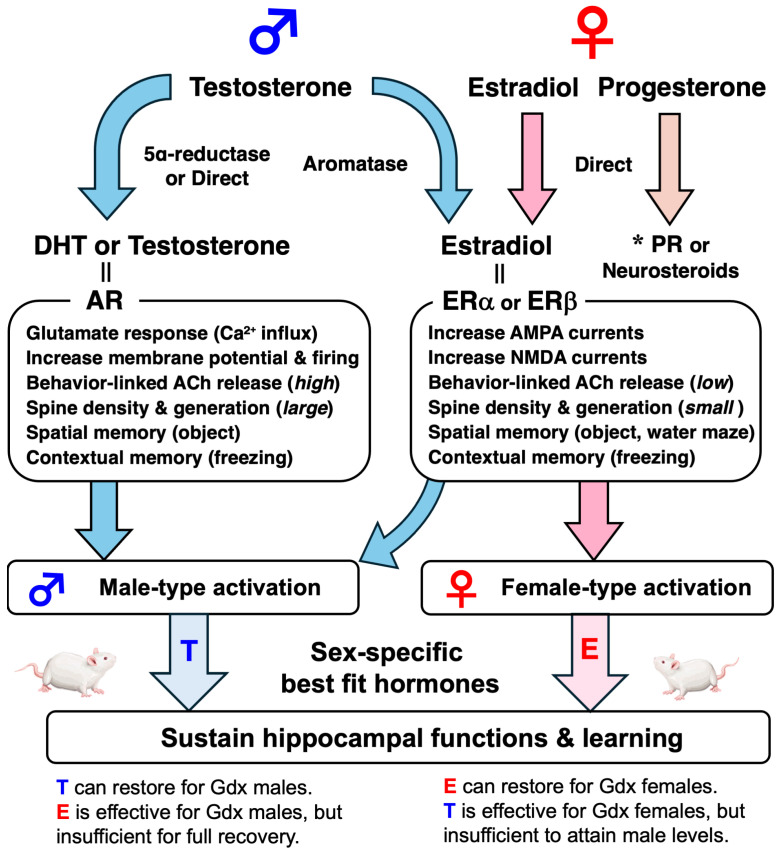
The activating effects of sex hormones on hippocampal function. In males, testosterone is metabolized by 5α-reductase to DHT or by aromatase to estradiol (E). Androgen receptors mediate the maintenance of spontaneous firing and dendritic spine density in CA1 pyramidal neurons. In females, estrogen receptors mediate the maintenance of AMPA/NMDA currents and dendritic spine density in CA1 pyramidal neurons. Although sex hormone levels are low in juveniles, testosterone strongly activates hippocampal function in males after puberty. This figure is based on published studies [15,22,23,24,25,32,33,34,35,36,37,38,39,40]. * The effects of progesterone and its metabolites are also important. T: testosterone, E: estradiol, DHT: dihydrotestosterone, AR: androgen receptor, ER: estrogen receptor, PR: progesterone receptor, ACh: acetylcholine, Gdx: gonadectomized.

After sexual maturation, the differential roles of steroid hormones such as estrogen and testosterone in regulating hippocampal function contribute to sex differences in contextual learning (Figure 3). In female rats, gonadectomy impairs contextual learning, but estrogen replacement restores hippocampal ACh release, reorganizes hippocampal circuitry, and improves learning [15,24,25]. Estrogen binds to estrogen receptor subtypes (ERα or ERβ), which are abundantly expressed in the hippocampus, leading to the activation of intracellular MAPK/ERK1/2 and PI3K/Akt signaling pathways [26]. These pathways enhance the expression of brain-derived neurotrophic factor (BDNF), which supports synaptic growth, dendritic spine density, and hippocampal-dependent memory formation [27]. Estrogen has also been shown to directly regulate the trafficking of AMPA and NMDA receptors, ensuring efficient excitatory synaptic transmission [28].

The hippocampus contains both 5-alpha reductase and aromatase, which convert testosterone to dihydrotestosterone (DHT) or estradiol [29,30]. Testosterone and its metabolites, including dihydrotestosterone and estradiol, can activate both estrogen and androgen receptors, affecting hippocampal structure and function through organizing effects during early development and activating effects after the onset of puberty [31]. Although gonadectomy impairs contextual learning in male rats, testosterone or androgen receptor agonist replacement restores hippocampal ACh release, increases resting membrane potential and firing, reorganizes hippocampal circuitry, and improves learning [15,22,23,32,33]. Estrogen replacement has some effects but not enough to restore ACh release and spine density to intact levels [15,32]. These findings suggest that males rely more heavily on androgen receptor-mediated mechanisms for spatial learning and memory tasks that become more apparent during adolescence and adulthood.

**Figure 4:**
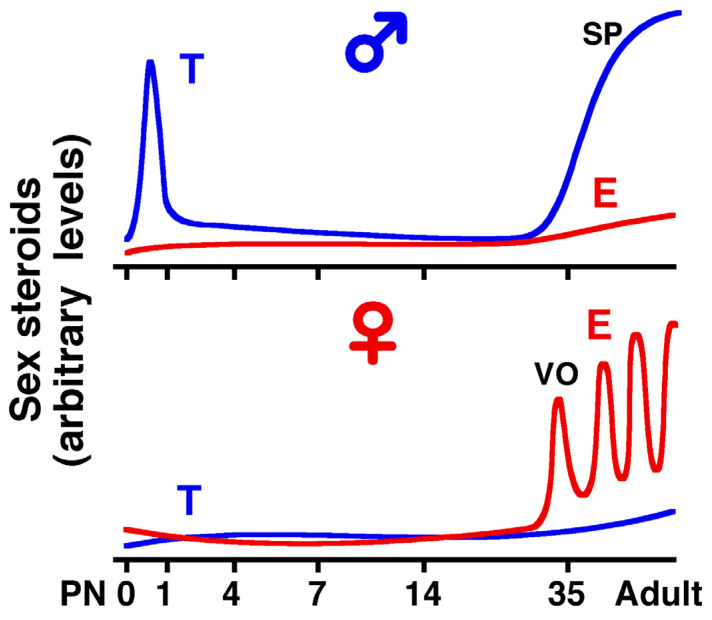
A diagram showing the developmental changes in serum sex hormone levels in male and female rats. The neonatal testosterone surge is known to fate the masculinization of the brain, although sex differences in circulating sex steroids were not clearly different until the fourth postnatal week. The day of birth is defined as PN0. T: testosterone, E: estradiol-17β. The timing of vaginal open (VO) and the detection of testicular spermatozoa (SP). This schema was drawn based on the published papers [42,43]. Radioimmunoassays were used to measure serum steroid levels.

Thus, after maturation, estrogen in females and testosterone in males are responsible for maintaining hippocampal function. More importantly, even when testosterone levels in gonadectomized females are supplemented up to the level of normal males, the brain does not become masculinized. Conversely, even when estrogen levels in gonadectomized males are supplemented up to the level of proestrus females, female characteristics are not reproduced. Although the developmental processes are still unknown, neonatal testosterone affects the sexual fate of the brain (Figure 4). When testosterone is artificially administered to female neonates, they reproduce male-specific patterns of hippocampal ACh release and sexual behavior. Conversely, when testosterone is blocked in neonatal males, they can induce LH surges through estrogen treatment [15,41].

### 4.3. Implications for Brain Sexual Differentiation

By blocking the masculinization of the brain that begins at PN0, we may be able to elucidate the primary cause of sex differences. Since brain masculinization does not occur in females, the activating effect of estrogen may have occurred earlier in females, leading to improved performance on the IA task in the juveniles. On the other hand, the organizing effect of testosterone may have delayed maturation in males, and their learning performance was relatively immature at PN16. However, careful and comprehensive behavioral assessment in both sexes is essential, as performance on the IA task and contextual fear conditioning are influenced by possible differences in pain sensitivity, motor ability, and emotional state [44].

The sex-specific developmental trajectory of hippocampal function is still unknown, but the faster development of learning functions in female infants suggests that female infants have shorter infantile amnesia. Further experiments are needed to determine the extent of this shorter period. The faster development of hippocampal function in females may have purposeful importance, helping to establish mother–child relationships in the next generation by remembering the mother’s parenting behaviors. As evidence of this, cross-fostering studies have already shown that infants cared for by mothers with high levels of parenting behaviors are more likely to have high levels of parenting behaviors themselves when they become mothers [45].

## 5. Conclusions

In the contextual learning task, juvenile females outperformed juvenile males, suggesting that females have a shorter period of infantile amnesia than males. Although the detailed developmental trajectory remains a mystery, hippocampal function develops steadily in females, whereas maturation may be delayed in males due to the organizing effects of neonatal testosterone that masculinizes the brain.

## Figures and Tables

**Table 1:** Postnatal groups and body weight.

Postnatal Days	Body Weight of Males	Number of Males	Body Weight of Females	Number of Females
16	35.2 ± 1.2 g	19	33.2 ± 0.8 g	21
17	38.3 ± 1.5 g	16	33.9 ± 1.4 g	8

## Data Availability

All raw data from this study are available from the authors upon request. The data are not publicly available due to the inclusion of data for ongoing research.
